# Research on the influence mechanism of employees’ innovation behavior in the context of digital transformation

**DOI:** 10.3389/fpsyg.2022.1090961

**Published:** 2022-12-20

**Authors:** Jiale Wu, Xiheng Gong, Yijun Liu

**Affiliations:** School of Economics and Management, Shanghai Institute of Technology, Shanghai, China

**Keywords:** digital transformation, employee innovation behavior, Grounded theory, Gioia method, interpretative structural model

## Abstract

With the booming of digital economy, digital transformation has become an important initiative for the high-quality development of enterprises, however, in the process of digital transformation, the innovation performance of most enterprises will be reduced, and employee innovation is the basis of enterprise innovation. How to improve employee innovation ability and guarantee the continuous innovation of the enterprise becomes the key issue. By conducting expert interviews and data crawling, the study uses Grounded theory and Gioia method to sort out the influencing factors of employee innovation behavior in digital transformation enterprises and use the interpretative structural model to analyze the complex relationships among the influencing factors, and establish a hierarchical structural model to organize and hierarchize them. The study shows that: leadership style, organizational innovation level, and organizational social responsibility are the fundamental influencing factors, emotions and personality traits are the direct influencing factors, and innovation expectations and innovation support feedback are important undertaking factors. The above findings provide theoretical guidance for companies to motivate employees to innovate and improve their innovation performance in the process of digital transformation.

## 1 Introduction

“The Outline of the Fourteenth 5-Year Plan for National Economic and Social Development and Vision 2035” emphasizes “accelerating digital development and building a digital China,” with special emphasis on building new advantages in the digital economy and promoting the digital transformation of industries. Faced with the volatility, uncertainty, complexity and ambiguity in digital transformation, how to stabilize innovation performance in the process of digital transformation has become a new challenge for the survival and development of enterprises, and employees, as a member of enterprises, bear an unshirkable responsibility for sustainable development. Employee innovative behavior (EIB) is the autonomous formation, promotion, and implementation of effective and novel ideas by employees of a company in their daily work to benefit their role performance, the group, and the organization (Janssen, 2000). It has been pointed out that employee innovation is the basis of enterprise innovation, and their unique resources, capabilities and creativity are the source of sustainable competitive advantage (Christina, 2004), and about 80% of new ideas in companies are proposed by employees (Isaac and Alan, 2003). Employee innovation behavior can not only effectively improve employees’ own innovation ability and innovation consciousness, but also help the enterprise to take the leading position in industry innovation and provide guarantee for the future development of the enterprise. Therefore, it is very important to explore which internal and external factors influence employee innovation behavior on the path of digital transformation of enterprises, in order to improve employee innovation behavior as well as enterprise innovation performance.

At the individual level, a study by Qiu et al. (2022) found that employees’ self-differentiation trait has a positive impact on their innovative behavior, and employees with a high degree of this trait don’t panic and are calm and collected, which can effectively motivate their innovative behavior to a certain extent. Isen (2009) found that positive emotions help to improve cognitive abilities, flexibility in perceiving things, and creative thinking to solve problems, while good emotions also positively influence work attitudes. On the contrary, when individuals are in a negative mood, they are usually unmotivated to settle for the *status quo* and their personal thinking and reactions become slower, which is not conducive to the generation of innovative ideas. The research of Rodell and Judge (2009) shows that different kinds of pressure will cause employees to have different emotions such as caring, anger and anxiety, and these different emotions will prompt employees to have different work behaviors. Knowledge acquisition mainly emphasizes the process in which employees acquire knowledge from outside to make up for their own knowledge stock and quality deficiency (Li et al., 2017). Innovation is the spiral of interaction between tacit knowledge and explicit knowledge, as well as the recombination and creation of knowledge (Men et al., 2021). In order to constantly meet the needs of innovation, employees must supplement and improve the knowledge reserve through learning or cooperation and communication with other knowledge sources (Drees and Heugens, 2012). Knowledge acquisition can break through the “familiarity trap” of innovation. Employees’ own knowledge is often too limited and narrow, which restricts the possibility of individual innovation. Knowledge acquisition means the sharing and integration of more cutting-edge theories and different viewpoints, which is conducive to employees’ obtaining more innovative inspiration and accelerating the generation of innovative behaviors (Mike et al., 2012). At the interpersonal level, a study by Rodell and Judge (2009) state that employees are exposed to different sources and types of stress at work, and their emotions are affected by stress, which in turn leads to positive or negative slackening work behaviors. And work pressure can be divided into challenging pressure and obstructive pressure (Cavanaugh et al., 2000). Leadership styles of leaders have an impact on employees’ innovative behaviors, and through literature combing, family supportive leadership (Wang et al., 2020), paradoxical leadership (Peng et al., 2020), inclusive leadership (Wu et al., 2020), servant leadership (Zhang and Zhu, 2021), ethical leadership (Zhang et al., 2021), benevolent leadership (Lu et al., 2021), authentic leadership (Xu et al., 2022), and coaching leadership (Song and Gao, 2020) all have significant impact on employees’ innovative behaviors. Choi (2012) found that colleague support enhances employee organizational identity, and employees will demonstrate behaviors consistent with organizational expectations through self-motivation, such as increased innovation and participation in innovative activities (Yu et al., 2021). Within a team or organization, breaking the barriers between the original knowledge owners can make knowledge flow freely to a certain extent, and the process of knowledge sharing can improve the innovative behavior and performance of the team or organization (Holub, 2003). Lin (2007) also pointed out that employees’ willingness to share knowledge with colleagues and to absorb and to learn knowledge from colleagues will help improve their innovation ability. The leader’s expectation of innovation will make employees tend to identify themselves as a creative person, and thus accept and voluntarily engage in corresponding innovative behaviors (Farmer et al., 2003). When employees perceive the leader’s expectation of innovation, in order to maintain a positive impression of consistency and support from the leader, they will generally push themselves to conduct exploratory or exploitative learning (Liu et al., 2021). On the other hand, leaders’ expectation, support and protection of innovation will create an atmosphere in the organization where people dare to challenge, take risks and learn by trial and error, so as to encourage active employees to take the initiative to challenge difficult problems and engage in creative work (Sun et al., 2012). At the organizational level, existing studies have shown that different types of organizational climate can significantly affect employees’ innovative behavior, such as employment relationship climate (Cao et al., 2021), error management climate (Jia et al., 2020), and organizational innovation climate (Li and Li, 2022). At the same time, organizational culture has long been regarded as an important means for organizations to integrate internal processes and adapt to the external environment. External adaptability focuses on the problems reflected externally, while internal integration focuses on the establishment of common vision and shared values (Denison and Mishra, 1995). Organizational culture is a collective phenomenon that affects the behavior of organizational members, as well as an important environmental factor that promotes the innovative behavior of employees (Yang et al., 2012). Different types of organizational culture will lead to different organizational performance and employee attitudes, thus affecting the innovative behavior of employees. In this regard, many scholars have elaborated the relationship between organizational culture and innovation (Sun et al., 2004; Lei et al., 2006; Wang and Wang, 2021).

Compared with existing studies, this paper makes the following two innovations: firstly, in terms of research perspective, the relationship between multiple influencing factors of employee innovation behavior in digital transformation enterprises is explored from three levels: individual–interpersonal–organizational, and research related to employee innovation behavior is conducted from a multidimensional perspective. This paper incorporates three influencing dimensions, such as digital work ability, digital innovation attitude and other important factors in the context of digital transformation, to study the influence mechanism of employee innovation behavior in the context of digital transformation from a refined perspective. Secondly, in terms of research method, this paper uses the Grounded theory and Gioia method to extract the factors that influence employees’ innovation behavior in the context of digital transformation, and uses the interpretative structural modeling (ISM) method to analyze the relationship between each influencing factor and the influence of each factor on employees’ innovation behavior. While collecting primary data, this study will also continuously supplement the secondary data to ensure that each important factor is rooted in the data and the whole model is supported by objective data as evidence. Meanwhile, on this basis, based on the interview results of experts and literature combing, the recursive structure model diagram of this study is finally determined, which enriches the research related to employee innovation behavior under digital transformation enterprises.

## 2 Research design

### 2.1 Research methods

#### 2.1.1 Grounded theory and Gioia method

In Glaser et al. (1968) established the research method of Grounded theory, which is used to develop new theories and summarize new cognition and understanding with phenomena. Therefore, it is also regarded as the most authoritative and normative qualitative research method. A comparison of the researcher’s way of thinking and practical process reveals that general quantitative empirical research adopts a top-down model, in which theoretical deductions and hypotheses are first proposed, then definitions and operations are constructed, and variable measurements are carried out after the definition of variables is derived; the rooted-theoretical research approach; on the other hand, adopts a bottom-up model, which does not require the formulation of corresponding theoretical hypotheses before the research is carried out, but takes the research question as the core and conducts the original data collection with practical observation. It does not need to put forward the corresponding theoretical hypothesis before the research is conducted, but takes the research question as the core, collects the original data by practical observation, and conducts in-depth thinking and summarization in the process, then tries to define the concept, gradually strips out the corresponding concepts and categories through three-level coding, and initially constructs the theoretical framework accordingly, and finally sorts out the main story line after the theory is revealed, and uses the condensed concepts and categories to establish the correlation between the elements, so as to get The corresponding theoretical perspective is formed.

Through the Grounded theory approach, Ding interviewed 27 expatriate employees to deeply explore the factors of expatriate motivation among employees of Chinese multinational operating companies and constructed a theoretical framework for the formation mechanism of expatriate motivation. The study pointed out that expatriate motivation can be divided into two categories based on the degree of self-determination: self-developmental motivation and duty-driven motivation, which are not opposed to each other but are on a continuum. The study expands the application of motivation theory in the field of expatriation and provides theoretical guidance for companies to effectively identify, motivate, and manage expatriate employees (Ding et al., 2022). Wang and Zeng (2022) use the Grounded theory approach, through in-depth interviews, to build a model of how the discourse in live web marketing affects the audience, extracting the four core categories of anchor level, product level, discourse chaos level, and audience level. The study pointed out that focusing on the regulation of the applicability of live discourse associated with anchor personalities, product information, and discourse chaos to make it work well for viewers and regulate live web marketing can promote reasonable consumption and achieve win-win situation for anchors, businesses, and consumers to promote social and economic development (Wang and Zeng, 2022).

The Gioia method (Mao, 2020) is essentially both phenomenologically driven and an iterative process of comparing data with theory. To avoid the researcher’s personal subjective bias, data analysis should usually involve two or more researchers analyzing the interview transcripts separately and independently looking for regular similarities and differences, after which agree on the categorization of the interview data (interviewees’ words) (forming first-order concepts), and then form themes (second-order) and convergent constructs. Themes (second-order) and convergent concepts. The body of the paper should fully cite primary data in order to flesh out the analytical presentation, and the data structure should present both persuasive evidence and shows the chain of data to theory. The Gioia method (Bamberger, 2018) systematically applies existing theories (including those outside the field of management) to help explain the data. The entire research process included data collection and analysis, review of relevant literature, and writing of theoretical notes. This is the more common rooted-theory approach, which involves iterations of three types of work: data collection and analysis, review of relevant literature, and writing theoretical notes. There are three objectives, namely, to find consistency with existing theory The objectives are to explore areas of agreement with existing theory, to highlight areas of conflict between data and theory, and to discover new concepts in order to construct new theories or new explanations of phenomena.

Li et al. (2022) conducted a rooting analysis of the collected and aggregated case materials based on the Grounded theory, presented the data structure using the Gioia method, and built a value creation mechanism model for digitally enabled advanced manufacturing enterprises by combining the second-order themes and aggregation constructs summarized in the collation. Gioia et al. (2013) used the Gioia method to show the process of personal media image change after being influenced by data structure diagrams as well as retrospective cause diagrams.

#### 2.1.2 Interpretative structural model

The ISM method is a widely used systems science method. It is derived from structural modeling (Liu, 2017), where the system to be analyzed is first divided into various subsystems (factors and elements) by combing, then analyzing the factors and the direct binary relationships between them; and mapping this conceptual model into a directed graph, and finally revealing the structure of the system through Boolean logic operations, and giving the overall function of the system without loss. The premise is presented as a minimalist hierarchical directed topological diagram (Chang and Wang, 2017). ISM has a great advantage over tables, text, mathematical formulas, and other ways to describe the essence of the system. Because it presents the conclusions in a hierarchical topological diagram, this presentation has an intuitive effect, and the hierarchical diagram allows one to understand at a glance the causal hierarchy of the system factors, the ladder structure.

In order to promote knowledge transfer within prefabricated construction enterprises and improve collaborative work efficiency, Shen builts a hierarchical structure model of influencing factors of knowledge transfer within prefabricated construction enterprises based on the theory of explanatory structural model. The results show that the transfer intention and organizational structure are the lowest factors and have the strongest driving force on the whole structural system (Shen et al., 2022). By analyzing the influencing factors of mobile social media burnout, Wan et al. (2022) used the explanatory structural model (ISM) and the cross-influence matrix multiplication method (MICMAC) to sort out the relationships of the influencing factors of mobile social media burnout and construct the corresponding mechanism models, and then proposed corresponding management strategies to provide management decision references for relevant enterprises and service operators to face and prevent mobile social media burnout crisis.

A review and comparison of the literature reveals that existing studies have explored the unilateral influences on employees’ innovative behavior in terms of individual factors (personality constitution, stress, etc.) and environmental factors (leadership style, colleague relationships, etc.), while some scholars have combined one of these influences or a few of them at different levels. However, individual behavior is the result of the interaction of multiple factors, and few scholars have studied the relationship between the factors influencing employees’ innovative behavior. In this paper, we use the Grounded theory and Gioia method to summarize and identify the factors that affect the innovation behavior of employees in digital transformation enterprises by conducting expert interviews and secondary data crawling, and analyze them from three levels: individual, interpersonal and organizational, using the explanatory structural model to establish a hierarchical recursive structural model, so as to provide theoretical guidance for enterprises to improve employees’ innovation ability and enterprise innovation performance in the process of digital transformation.

### 2.2 Selection of research subjects

This research selects the sample in strict accordance with the following principles: (1) the selected digitally transformed companies have sufficient relevant information, and the employee innovation process is clear and distinct in stages; (2) they have certain R&D advantages, rely on technological scalability and innovation to provide new products and services, and aim at sustainable development; (3) employees are involved in the innovation reconstruction and value creation change process of the company.

Based on the above criteria, this paper refers to the “White Paper on China’s Top 500 Advanced Manufacturing Brands” and related industry reports for theoretical sampling, with the principle of theoretical saturation, i.e., adding, comparing and evaluating the degree of theoretical saturation of the sample, finally 15 enterprises were selected for this study, and 10 enterprises were randomly selected as the original information for coding, and the remaining five enterprises were used as the saturation test information for the results of the rooted theory refinement.

### 2.3 Data collection process

For the sake of improving the value of data utilization and ensuring the authenticity of data, this study adopted a composite channel to collect different types of primary data with different attributes, including semi-structured interviews, fieldwork, and expert consultation, in order to obtain detailed primary data. After collecting the primary data, this study divided the researchers into two groups, and the two groups of researchers simultaneously reviewed the content of the collected recordings and texts, and organized and recorded them by textual means; Finally, the two groups will compare and analyze and discuss the content of the review. If the researchers have different opinions or ambiguous text expressions during the discussion, then they will conduct interviews with the enterprise data providers again around the issues that arise to ensure that the original data is correct before conducting data analysis. In addition, in order to enrich the research data and avoid common methodological bias, this study collected primary data and searched for “digital transformation” and “employee innovation behavior” on China National Knowledge Infrastructure, CQVip, Tongfang, and China Academic Survey Database to fully supplement the secondary data. In the process of collection, the principle of problem orientation was always maintained, and a large amount of relevant data was collected. Finally, the collected data are labeled and summarized as first-order concepts, while the relevant second-order themes are summarized and condensed based on the text content and reading literature, and the final aggregated constructs are analyzed to ensure that the information truly reflects the influencing factors of employees’ innovative behaviors for the subsequent research as well as discussion.

In this research, both entrepreneur data and employee data are collected. The entrepreneur data are mainly obtained through interviews and inspections; the employee innovation behavior data are obtained in the following ways: (1) semi-structured interviews with employees of enterprises in the downstream of the industrial chain of the sample enterprises to obtain primary data; (2) crawling employee-related information in the digital platforms of major enterprises to obtain secondary data; (3) random telephone interviews with employees of enterprises to obtain primary data.

## 3 Data organization and analysis

### 3.1 Data structure diagram

Gioia method belongs to the more common Grounded theory method. The advantage of this method is that the evidence is well presented, the evidence chain is solid, and each important finding is rooted in data, including from first-order concepts to second-order themes and aggregate dimensions, and each first-order concept has original data examples, so it gives a very strong sense of heaviness in evidence presentation and is suitable for doing rigorous qualitative research and analysis.

**FIGURE 1:**
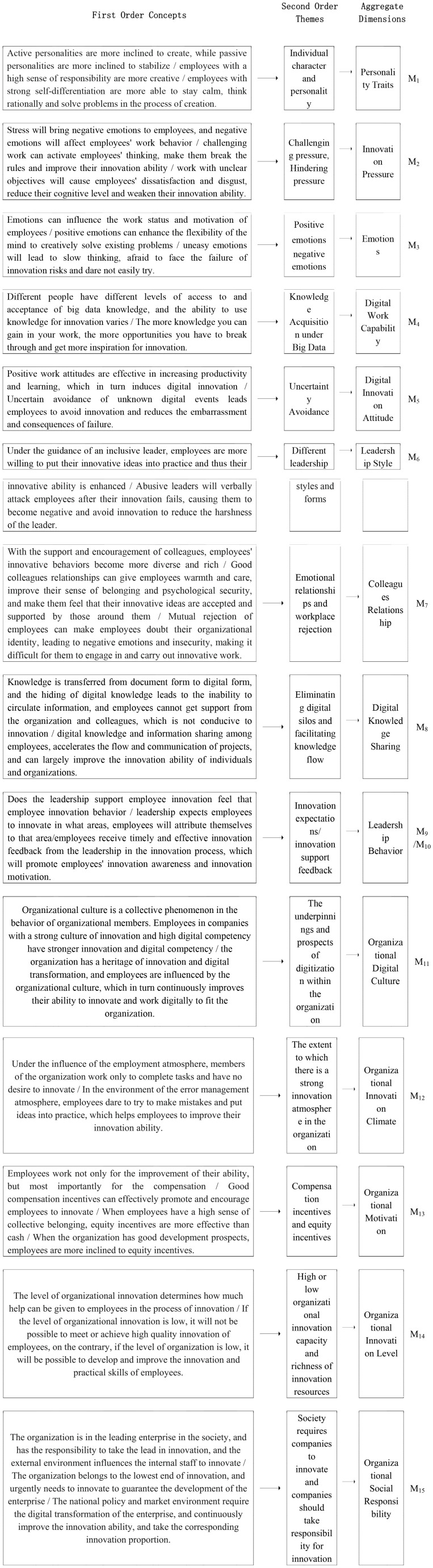
Data structure of factors influencing employees’ innovation behavior.

In this paper, 35 first-order concepts, 19 second-order themes, and 14 aggregate dimensions were summarized by induction of the original information collected (Li et al., 2022), and the specific data structure diagram is shown in Figure 1.

### 3.2 Theoretical model construction

**FIGURE 2:**
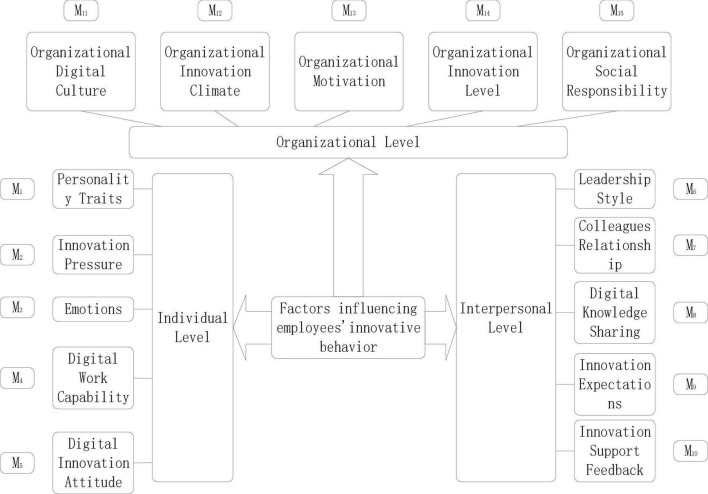
Theoretical model of factors influencing employees’ innovative behavior.

In this study, through the work of organizing first-order concepts, merging second-order themes, and refining aggregate dimensions, we found that the factors influencing employees’ innovative behavior contain three levels, which are individual level, interpersonal level, and organizational level; At the same time, combined with related literature, research reports, and some primary data, we construct a theoretical model of factors influencing employees’ innovative behavior, as shown in Figure 2.

### 3.3 Theoretical saturation test

In this paper, a total of 15 companies suitable for this paper’s study were selected for semi-structured interviews and field trip. Through analysis and comparison, and 10 companies’ interview data were used for analysis to derive a certain number of influencing factors, while the remaining five companies’ interview data were used for theoretical saturation tests according to the research process of Grounded theory, but no new important categories or concepts emerged. Clearly, the categories at all levels have been developed more richly and the theoretical model has been saturated in this study (Zhang M. et al., 2017).

### 3.4 ISM analysis of employee innovation behavior in the context of digital transformation

By numbering the factors influencing employees’ innovative behavior derived from Grounded theory and Gioia method, and conducting semi-structured interviews with six experts, three of whom are experts in organizational behavior in universities and have participated in the evaluation and scoring of EIB for many times, and the other three experts are highly educated talents in their enterprises, with rich experience in enterprise innovation management and talent innovation training. If more than half of the experts think that *M*_i_ directly affects *M*_j_, then *M*_ij_ = 1, and if not, *M*_ij_ = 0. Accordingly, the adjacency matrix of the factors influencing employees’ innovative behavior was obtained. Meanwhile, according to the actual situation of the interview, the progress and questions are adjusted in time to ensure the reasonableness and authenticity of the interview data, and finally the factors characteristics and experts’ opinions are combined to establish the connection for the factors influencing employees’ innovative behaviors, and finally the adjacency matrix of two elements is formed, as shown in Table 1.

**TABLE 1:** Adjacency matrix.

	M_1_	M_2_	M_3_	M_4_	M_5_	M_6_	M_7_	M_8_	M_9_	M_10_	M_11_	M_12_	M_13_	M_14_	M_15_
M_1_	0	0	0	0	0	0	0	0	0	0	0	0	0	0	0
M_2_	0	0	0	0	1	0	0	0	1	0	0	0	0	0	0
M_3_	0	0	0	0	0	0	0	0	0	0	0	0	0	0	0
M_4_	0	0	1	0	0	0	0	0	0	0	0	0	0	0	0
M_5_	1	0	1	0	0	0	0	0	0	0	0	0	0	0	0
M_6_	0	0	0	0	0	0	1	1	0	0	1	0	0	0	0
M_7_	0	1	0	0	0	0	0	0	0	1	0	0	0	0	0
M_8_	0	1	0	0	0	0	0	0	0	1	0	0	0	0	0
M_9_	1	0	1	0	0	0	0	0	0	0	0	0	0	0	0
M_10_	0	0	0	1	0	0	0	0	1	0	0	0	0	0	0
M_11_	0	1	0	0	0	0	0	0	0	1	0	1	0	0	0
M_12_	0	0	0	0	1	0	0	0	1	0	0	0	0	0	0
M_13_	0	0	0	0	0	0	0	0	0	0	0	1	0	0	0
M_14_	0	0	0	0	0	0	0	0	0	0	1	0	1	0	0
M_15_	0	0	0	0	0	0	0	0	0	0	1	0	1	0	0

Based on the adjacency matrix in Table 1, the corresponding reachability matrix is calculated in Matlab2016a, as shown in Table 2. Where *M*_ij_ indicates whether the influence factor *M*_i_ is reachable to *M*_j_, and if it is reachable, then *M*_ij_ = 1, and if it is not reachable, then *M*_ij_ = 0.

**TABLE 2:** Reachability matrix.

	M_1_	M_2_	M_3_	M_4_	M_5_	M_6_	M_7_	M_8_	M_9_	M_10_	M_11_	M_12_	M_13_	M_14_	M_15_
M_1_	1	0	0	0	0	0	0	0	0	0	0	0	0	0	0
M_2_	1	1	1	0	1	0	0	0	1	0	0	0	0	0	0
M_3_	0	0	1	0	0	0	0	0	0	0	0	0	0	0	0
M_4_	0	0	1	1	0	0	0	0	0	0	0	0	0	0	0
M_5_	1	0	1	0	1	0	0	0	0	0	0	0	0	0	0
M_6_	1	1	1	1	1	1	1	1	1	1	1	1	0	0	0
M_7_	1	1	1	1	1	0	1	0	1	1	0	0	0	0	0
M_8_	1	1	1	1	1	0	0	1	1	1	0	0	0	0	0
M_9_	1	0	1	0	0	0	0	0	1	0	0	0	0	0	0
M_10_	1	0	1	1	0	0	0	0	1	1	0	0	0	0	0
M_11_	1	1	1	1	1	0	0	0	1	1	1	1	0	0	0
M_12_	1	0	1	0	1	0	0	0	1	0	0	1	0	0	0
M_13_	1	0	1	0	1	0	0	0	1	0	0	1	1	0	0
M_14_	1	1	1	1	1	0	0	0	1	1	1	1	1	1	0
M_15_	1	1	1	1	1	0	0	0	1	1	1	1	1	0	1

**FIGURE 3:**
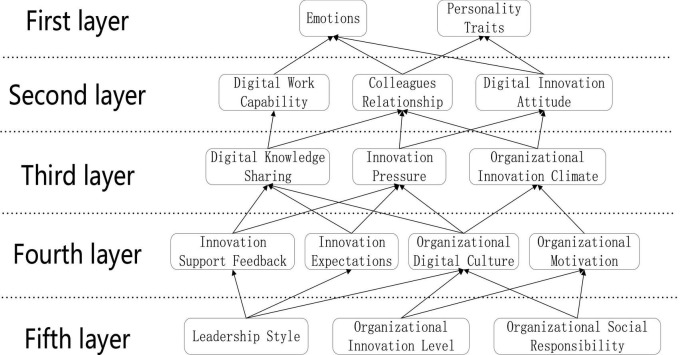
Recursive structural relationship model of factors influencing employees’ innovation behavior.

Through the reachability Matrix in Table 2, the hierarchical combing is continuously carried out, and finally the results of the hierarchical division of the factors influencing employee innovation behavior are obtained, and the two-by-two relationship between the factors in the adjacency matrix is used to construct the recursive structural relationship model of the factors influencing employee innovation behavior in this study, as shown in Figure 3.

As can be seen from Figure 3, the factors influencing employees’ innovative behavior in the context of digital transformation can be divided into five layers, which can be divided into fundamental, intermediate and apparent layers according to the explanatory structural model method, where the fifth layer is the fundamental layer, the third layer and fourth layer are the intermediate layer, and the first layer and second layer are the apparent layer (Lin, 2007).

#### 3.4.1 Fundamental layer

The level of organizational innovation is a fundamental influencing factor that provides resource constraints for employees to achieve high levels of innovation in the digital transformation process. The level of organizational innovation will influence the innovation consciousness and innovation ability of enterprise employees, and prompt changes in the innovation performance of the whole enterprise to transform and upgrade from a low level innovation enterprise to a high level innovation enterprise. Leadership style and organizational social responsibility are the most fundamental factors influencing employees’ innovative behavior and are among the deepest causes in the ISM model. We should strive to cultivate positive and inclusive leaders, actively assume due social responsibility, strengthen social cooperation, and learn from the experiences of successfully transformed companies to provide a reference base for the realization of digital transformation.

#### 3.4.2 Intermediate layer

The influencing factors in the fourth layer are innovation support feedback, innovation expectations, organizational digital culture, and organizational motivation; and the influencing factors in the third layer are digital knowledge sharing, innovation pressure, and organizational innovation climate. Innovation support feedback and innovation expectations can make it clear to employees whether their innovation behaviors are supported or not. Depending on the level of support and expectations, employees will have different degrees of pressure and motivation in the innovation process, and also influence the digital knowledge sharing behavior among employees to a certain extent. If an organization has a good sharing culture in the digital era, then it will motivate employees to share digital knowledge and form a good and harmonious working atmosphere. A sound organizational motivation system can effectively improve the motivation of employees to innovate and form a work atmosphere where you can catch up with me.

#### 3.4.3 Apparent layer

The second level of influences are digital work ability (knowledge acquisition), colleagues’ relationship, and digital innovation attitudes (uncertainty avoidance); the first level of influences are emotions and personality traits. In organizations, the ease of knowledge acquisition can influence employees’ emotions in the process of knowledge acquisition, which in turn affects their innovation behavior. A good emotional relationship between co-workers can enhance the positive emotions of employees at work. If there is rejection or isolation among co-workers, then the isolated employee will fall into self-doubt and have a certain degree of negative impact and change on personal traits. Uncertainty avoidance attitude toward innovation will cause employees to have self-doubt and question their own ability, which will have an impact on individual emotions. As the number of avoidance innovation behaviors increases, employees’ personal traits will also be affected, and they will gradually develop from bold innovation to conservative, which is not good for employees’ innovation and enterprise development.

Through the Grounded theory and Gioia method, it is known that employees’ innovative behaviors are influenced by internal and external factors (individual, interpersonal, and organizational). However, it is unknown which of these factors have a greater impact on employees’ innovation behavior and which have a smaller impact on their innovation behavior in the process of digital transformation. These influencing factors can be divided into the fundamental layer, the intermediate layer, the apparent layer. From the fundamental layer, organizational social responsibility can urge enterprises to take the initiative to assume corresponding responsibilities, strengthen social cooperation and continuously innovate. While the basis of an enterprise’s development is its level of innovation, and a high level of innovation can provide solid backing for leaders and employees in the enterprise, while a highly inclusive leader will also provide a strong boost when employees need material and spiritual help; From the intermediate layer, both pressure (innovation pressure, innovation expectations, etc.) and motivation (organizational motivation, digital knowledge sharing, etc.) can effectively increase employees’ desire to innovate and stimulate their innovative behavior, which can realize the value of self-innovation and create more development space for the enterprise at the same time; From the apparent layer, digital work ability, personality traits, and the changing environment around them will also constantly influence employees’ innovative behavior. With the digital changes in work ability requirements and work environment, if employees cannot adapt and make corresponding changes as soon as possible, then even if excellent innovative ideas emerge, they will be limited to the limitations of technology or ability and cannot be realized.

## 4 Discussion and conclusion

Studies have shown that employees’ personal perseverance can effectively improve their innovative behavior, and they will not flinch from difficulties and go forward to meet them (Liu et al., 2022). Some scholars have pointed out that challenging stressors can have a significant positive impact on employees’ innovative behaviors through dual mediating effects, and can effectively stimulate employees’ innovative behaviors (Fang et al., 2022). By studying the relationship between workplace exclusion behaviors among colleagues and employees’ innovation behaviors, Chen et al. (2022) found that workplace exclusion not only directly affects employees’ innovation behaviors, but also reduces employees’ innovation behaviors by affecting their emotions. Lin and Liu believed that leaders’ constructive feedback would have a positive impact on employees’ innovative behavior through their affected emotions (Lin and Liu, 2022). Chen pointed out that the innovative behavior of employees under the atmosphere of error aversion is significantly reduced (Chen, 2022), while the organizational innovation atmosphere can significantly improve the innovative behavior of employees (Li and Li, 2022). Friendly and humorous leadership can significantly improve employees’ innovative behavior, which is conducive to enterprise development (Wang and Wang, 2022). Zhou and Wang (2019) and Zhao and Wang (2021) found that employee innovation behavior was influenced by various factors. And the degree of influence of each factor on employee innovation behavior varied (Liu et al., 2020; Zhao and Xiao, 2021).

So, in this paper, from a system perspective, we study the mechanism of influencing employee innovation behavior in the context of digital transformation, and use the Grounded theory and Gioia method to sort out 15 factors that influence employee innovation behavior in the context of digital transformation, and use the explanatory structural model method to clearly and explicitly describe the logical relationship and the path of action between each factor. On this basis, the recursive structural model of employee innovation behavior in the context of digital transformation is constructed, and the influence mechanism of employee innovation behavior in the context of digital transformation is systematically elaborated. Firstly, leadership style, organizational innovation level, and organizational social responsibility are the most fundamental and critical elements affecting employees’ innovation behavior. Secondly, innovation expectations and innovation support feedback, etc., are the undertaking of direct and fundamental influencing factors. Finally, emotions and personality traits, etc., are the direct influencing factors.

A comparison of previous studies shows that employee innovation plays a crucial role in the survival and development of the organization, and that the leadership style of the managers themselves always influences employee innovation (Lin et al., 2022). Transformational leaders make employees feel the importance of work tasks, provide opportunities for subordinates, and empower subordinates (Alan et al., 1995) to meet the psychological needs of employees’ autonomy, which is conducive to stimulating subordinates’ intrinsic motivation to innovate; Inclusive leaders maintain an open attitude when interacting with subordinates, which is conducive to the generation of new ideas; Inclusive leaders’ acceptance of subordinates’ ideas is conducive to the promotion of innovation (Abraham et al., 2010). Lee and Chang (Sun and Henny, 2020) concluded that leadership style (charismatic inspiration) is significantly and positively related to employees’ innovation ability and has a greater impact on managerial innovation to improve employees’ skills than on R&D innovation to generate new methods or technologies; The external environment is a resource for employees in organizations to exercise prediction, manipulation or volitional control and self-reflection, and organizational innovation capability as an important external environment, enterprises continuously increase their support for innovation activities, such as increasing R&D investment and sending outstanding personnel to study overseas, which helps to create an organizational climate of innovation for all employees and gradually form an organizational culture that respects innovative ways of working (Wang, 2021); Ubius and Aals (2012) analyzed from the perspective of innovation climate and concluded that organizational social responsibility has a positive impact on the innovation climate of enterprises. Rexhepi et al. (2013) showed that companies that properly fulfill their social responsibility and place it at the height of their development strategy can effectively promote product and process innovation, which ultimately leads to long-term value enhancement. So, this paper argues that leadership style, organizational innovation level, and organizational social responsibility are the most fundamental and critical elements affecting employees’ innovation behavior.

Some scholars point out that superiors in an organization often exert influence on subordinates through feedback, that is, the information transmitted by the sender and related to the performance of the receiver (Zhang J. et al., 2017). Innovative feedback precisely reflects the trust and developmental expectations of superiors toward employees, it emphasizes that feedback in an encouraging and supportive manner will have a certain impact on employees’ innovative activities and promote their performance (Pamela, 2002). At the same time, scholarly research has shown that developmental feedback from superiors promotes employee innovation (Baek et al., 2012). Organizational culture is a collective phenomenon that affects the behavior of organizational members and is also an important environmental factor promoting employees’ innovative behavior (Yang et al., 2012). Many scholars have expounded the relationship between organizational culture and innovation, and found that harmonious and healthy organizational culture can have a positive impact on employees’ innovation (Sun et al., 2004; Lei et al., 2006; Wang and Wang, 2021). The relationship between organizational motivation as well as organizational constraints and innovation has received extensive attention from scholars (Byron and Khazanchi, 2012; Rosso, 2014). Research generally agrees that innovation is a process that requires extrinsic motivation, adequate resources, time and self-control. Motivation provided by the organization increases the employee’s sense of self-determination, which in turn stimulates intrinsic motivation; While factors such as lack of resources and time pressure can weaken intrinsic motivation and hinder innovation (Eisenberger and Rhoades, 2001). The process of knowledge sharing can enhance the innovative behavior and performance of the team or organization (Holub, 2003), and Lin (2007) also stated that employees’ willingness to share knowledge with colleagues and to absorb and learn from them will help to enhance innovation. So, this paper argues that innovation expectations and innovation support feedback, etc., are the undertaking of direct and fundamental influencing factors.

The research results of Yesil and Sozbilir (2013) shows that personality traits are positively correlated with individual innovation behavior. They believe that personality traits are one of the important factors affecting individual innovation ability and has great significance for individual innovation behavior in the workplace. Madrid et al. (2014) collected data from 92 different occupations employed by 73 different companies, proposed and tested a multi-level and interactive individual innovation model, and adopted a ring model of emotion. They believed that weekly positive and happy emotions were more conducive to stimulating innovative work behaviors. Knowledge acquisition in digital work capability can break the “familiarity trap” of innovation. Employees’ own knowledge is often too limited and narrow, which confines the possibility of individual innovation, while knowledge acquisition means the sharing and integration of more cutting-edge theories and different perspectives, which is conducive to employees’ acquisition of more inspiration for innovation and accelerates the generation of innovative behaviors (Mike et al., 2012). And in the study of uncertainty avoidance behavior in digital innovation attitudes, it has been noted that since the process of innovation is full of uncertainty, highly uncertain employees will avoid innovation, and employees with high uncertainty avoidance will feel anxious and uneasy in the face of uncertain innovation situations, exacerbating the fear of innovation failure, and thus affecting the process from innovative ideas to practice (Zhou and Song, 2020). Colleagues’ relationship refers to various communication activities between colleagues outside work to enhance the relationship (Wang et al., 2008). When the Colleagues’ relationship between colleagues reaches a high level, the innovative working ideas and working methods of employees are more likely to be understood and respected by the surrounding colleagues (Xiao and Peng, 2008). Meanwhile, such understanding and respect can also improve the psychological security perception of employees on their working environment (Teresa and Michael, 2016). Thus, it can stimulate employees’ innovation activities (Xu et al., 2019). So, this paper argues that emotions and personality traits, etc., are the direct influencing factors.

## 5 Research implications and recommendations

### 5.1 Theoretical contributions

First of all, the theoretical exploration and evidence related to the factors and pathways of influence on employee innovation behavior are supplemented by the use of the Grounded theory and the Gioia method. In the context of “mass entrepreneurship and innovation,” not only enterprises have to innovate themselves, but also employees have to participate in innovation, and there are many factors that influence employees’ innovative behavior in previous studies, including those that can positively promote employee innovation and those that hinder it, but fewer studies have examined whether there is an interaction between the various influences. By exploring the factors that influence employees’ innovative behavior, this paper can provide enlightening research evidence and a complete research framework for improving employees’ innovative behavior.

Second, the ISM method is used to construct a hierarchical progressive structure model of the factors influencing employee innovation behavior to show the direct factors, middle factors, and fundamental factors influencing employee innovation behavior in a more intuitive form to explain that employee innovation behavior is influenced by multiple factors, which further complements and expands the theoretical study of employee innovation behavior.

### 5.2 Practical implications

Firstly, in the context of the implementation of innovative country strategy, in today’s world of relying on innovation to win, the level of innovation ability directly determines the ability of an enterprise to maintain its leading position in the industry. Therefore, the spiritual trait of employee innovation is also getting more and more attention, and employees with high innovative behavior play an important role in promoting the innovative development of enterprises as well as social harmony. The study of the influencing factors of employee innovation behavior enables entrepreneurs to improve employee innovation behavior through scientific and proactive behaviors targeted at enhancing employee innovation behavior, which in turn strengthens the willingness of enterprises to sustain innovation and helps enterprises to invest in innovation and improve innovation capacity.

Secondly, in the context of dual innovation, innovation seems to have become the key to the success of enterprises, and the only way to break through the bottleneck period of productivity is to innovate in order to gain a more lasting vitality. However, innovation is not an easy task, and it requires the joint efforts of entrepreneurs and employees. How to stimulate employees’ innovative energy and motivate them to generate continuous and beneficial innovation has become an urgent problem for modern enterprises to face. Therefore, by studying the antecedent and causal variables of employees’ innovation behavior, this paper tries to provide new ideas for stimulating employees’ innovation behavior.

Thirdly, in the context of digital transformation of enterprises, if enterprises want to sustain long-term development, they cannot do without the proactive innovation of employees. Therefore, in this paper, we construct a hierarchical recursive model for entrepreneurs to select the most fundamental and critical factors that affect employees’ innovation behavior in order to improve employees’ innovation behavior and enterprise innovation performance.

### 5.3 Recommendations

In order to improve corporate innovation performance and employee innovation behavior in the process of digital transformation, based on the findings of this paper, the following three countermeasures and suggestions are proposed.

First, actively improve the level of organizational innovation. Actively respond to and implement national policies to create an atmosphere of positive corporate innovation. Under the call of the state to encourage digital transformation of enterprises, the introduction of digital technology reshapes the production process, organizational structure and business model of enterprises, changes the inherent management thinking logic, and drives enterprises to launch a full range of disruptive innovation. Enterprises establish a sound innovation incentive system to guide employees to participate in corporate innovation through digital learning and enhance their innovation capabilities.

Second, the role of the leader as a role model. Leaders themselves set an example by actively conducting digital learning and innovation training, improving their own innovation ability, practicing innovation behavior in their daily work, encouraging and guiding employees to actively participate in innovation training, improving their own innovation ability and level, and also giving verbal praise and material rewards to employees in the process of implementing innovation to motivate and guide them to innovate and enhance their innovation self-confidence and enthusiasm.

Third, pay attention to the emotional changes of employees in a timely manner. Factors such as employees’ personality traits and relationships among colleagues can have an impact on employees’ emotions. Each employee’s personality traits are different and determine how they respond in the face of corporate digital transformation. Managers can provide targeted and innovative guidance and training based on their traits, and reasonably assign work content to keep them in a positive emotional and work state. Colleagues, as teammates with whom employees spend time together, have a very important influence on employees’ work behavior. Friendly relations among colleagues can be promoted through group activities, company parties and other projects so that employees can work with colleagues in a positive mood for teamwork. Companies can also maintain the emotional stability of their employees by providing them with regular psychological counseling and de-escalation services.

### 5.4 Future outlook

The great changes in the global economic situation, the intensification of competition, the restriction of energy environment and the low cost of labor all restrict the innovation and development of enterprises. Both the external environment and the internal environment have an impact on enterprises and their employees to some extent. In the face of the increasingly fierce competition for resources, the reasonable allocation of resources within the enterprise is very important. Through the research on the influencing factors of employees’ innovation behavior in this paper, enterprises should pay more attention to the subject status of employees and give full play to their subjective initiative while paying attention to their own innovation level. From the point of view of the economic benefit of the enterprise, the employee is the main body of creating value. In the long run, employees are the cornerstone of a company’s continuous operation. In the future, the position of employees will be more important, and the research on employee behavior should be more in-depth.

## 6 Limitations and direction for future research

Due to the limitation of time and research ability, there are still numerous shortcomings in this paper to study the factors influencing employees’ innovation behavior. Firstly, the amount of data obtained from interview information is insufficient and the scope of interview subjects is small. The number of the subjects of the Grounded theory study, the information obtained from the webpage has certain limitations; secondly, the factors affecting employees’ innovative behavior are not comprehensive enough, and the accuracy of the analysis results may be affected to some extent because some fuzzy concepts have not been summarized and organized due to the sample data and other reasons; Finally, the data of this study come from the subjective reports with the subjects, and there may be certain errors (such as memory bias, Social approval, etc.), future related studies can expand the data collection channels and collect data from multiple sources, which may also measure the relevant variables more objectively.

## Data availability statement

The original contributions presented in this study are included in the article/supplementary material, further inquiries can be directed to the corresponding author.

## Author contributions

JW and XG designed the research, performed the research, analyzed the data, and wrote the manuscript. YL participated in revising the manuscript. All authors contributed to the article and approved the submitted version.
